# Antimetastatic gene expression profiles mediated by retinoic acid receptor beta 2 in MDA-MB-435 breast cancer cells

**DOI:** 10.1186/1471-2407-5-140

**Published:** 2005-10-28

**Authors:** Brett Wallden, Mary Emond, Mari E Swift, Mary L Disis, Karen Swisshelm

**Affiliations:** 1 Department of Pathology, Box 357470, University of Washington, Seattle, WA, USA; 2 Department of Biostatistics, Box 357232, University of Washington, Seattle, WA, USA; 3 Division of Oncology, Box 358050, University of Washington, Seattle, WA, USA

## Abstract

**Background:**

The retinoic acid receptor beta 2 (RARβ2) gene modulates proliferation and survival of cultured human breast cancer cells. Previously we showed that ectopic expression of RARβ2 in a mouse xenograft model prevented metastasis, even in the absence of the ligand, all-*trans *retinoic acid. We investigated both cultured cells and xenograft tumors in order to delineate the gene expression profiles responsible for an antimetastatic phenotype.

**Methods:**

RNA from MDA-MB-435 human breast cancer cells transduced with RARβ2 or empty retroviral vector (LXSN) was analyzed using Agilent Human 1A Oligo microarrays. The one hundred probes with the greatest differential intensity (p < 0.004, jointly) were determined by selecting the top median log ratios from eight-paired microarrays. Validation of differences in expression was done using Northern blot analysis and quantitative RT-PCR (qRT-PCR). We determined expression of selected genes in xenograft tumors.

**Results:**

RARβ2 cells exhibit gene profiles with overrepresentation of genes from Xq28 (p = 2 × 10^-8^), a cytogenetic region that contains a large portion of the cancer/testis antigen gene family. Other functions or factors impacted by the presence of exogenous RARβ2 include mediators of the immune response and transcriptional regulatory mechanisms. Thirteen of fifteen (87%) of the genes evaluated in xenograft tumors were consistent with differences we found in the cell cultures (p = 0.007).

**Conclusion:**

Antimetastatic RARβ2 signalling, direct or indirect, results in an elevation of expression for genes such as tumor-cell antigens (CTAG1 and CTAG2), those involved in innate immune response (e.g., RIG-I/DDX58), and tumor suppressor functions (e.g., TYRP1). Genes whose expression is diminished by RARβ2 signalling include cell adhesion functions (e.g, CD164) nutritional or metabolic processes (e.g., FABP6), and the transcription factor, JUN.

## Background

It is estimated that approximately 15–25% of women with node-negative breast cancer will eventually succumb to the disease due to distant metastases [1]. While important and promising anti-hormonal therapies – tamoxifen and aromatase inhibitors – exist for postmenopausal breast cancer patients with estrogen receptor positive tumors [2], chemo- and radiation-therapy remain the major options for all other women with progressive disease. Elucidating gene expression alterations in metastatic lesions compared to non-metastatic tumors is compelling, and orthotopic xenograft models provide an avenue for tackling this challenge. The results of such studies could lead to rapid translational research and drug discovery [3,4]. Several candidate metastasis-promoting proteins have been identified in differential screening of cell cultures and primary invasive tumors and these include ERB2/Her2/neu, VEGF and stromelysin [5]. An exciting new field of investigation, the search for proteins responsible for inhibition of the metastatic cascade, "metastasis suppressor" genes, has revealed a set of molecules that include: NM23 (histidine kinase), KAI1 (a tetraspanin integral membrane protein that responds to NFκB), BRMS1 (gap junction function), and MKK4 (mitogen-activated protein kinase) [4,6,7]. Functions regulated by metastasis suppressors include transcription, signal transduction, cell adhesion, and inflammation. We have demonstrated that RARβ2, with known tumor suppressor functions, also confers antimetastatic properties in a xenograft model of human breast cancer [8].

RARs are members of the nuclear hormone receptor superfamily, and in conjunction with their heterodimeric partners, the retinoid X receptors (RXRs), they positively or negatively regulate (most commonly) genes containing a direct repeat five (DR5) retinoic acid response element (RARE) within promoters. Transactivation via these heterodimeric partners is generally conferred by physiological or pharmacologic levels of retinoid-derived ligands, including all-*trans *retinoic acid (AT-RA) and 9-*cis*-retinoic acid, for RARs and RXRs, respectively. We and others have shown that RARβ2 or RARβ4 mRNA is diminished in most breast cancer cell lines [9,10]. Several laboratories have also demonstrated loss or diminished expression of the RARβ2 mRNA in primary breast cancers [11,12]. The mechanism(s) for diminished RARβ2 expression include transcriptional repression by epigenetic silencing [13-15]. A truncated, oncogenic RARβ protein (RARβ-prime) is exclusively expressed in breast cancer cell lines, and the presence of this isoform likely obstructs tumor suppressor functions of RARβ2 and RARβ4 protein isoforms [16,17].

RARβ2 activates both tumor suppressor and antimetastatic programs. In cell cultures in which RARβ2 has been introduced via a retroviral vector, we found that all breast cancer cells could be inhibited in their proliferative capacity, even in the absence of the natural ligand, AT-RA. Moreover, there is a dichotomy in RARβ2-transduced tumor cells in response to AT-RA: ER-positive cells undergo apoptosis, while ER-negative cells are further reduced in their proliferative potential, in comparison to culture conditions in the absence of AT-RA. In our xenograft model, we engrafted MDA-MB-435 breast cancer cells containing either LXSN-vector or LXSN-RARβ2 into the mammary fat pad (mfp) of nude or SCID mice [8]. Our model was designed to mimic the treatment of patients with advanced local disease. After 12–15 weeks, the mfp-encapsulated, primary tumors were fully resected. Following an additional 7–11 weeks, the animals were necropsied and analyzed for multiple parameters, including incidence of metastases – the most common sites being lungs and pleura. The overall incidence of metastasis was 37% (19 of 52 mice) in the vector-control animals compared to 1.8% (1 of 55 mice) in the RARβ2-expressing tumor cell implants (p < 0.00001). The one metastatic lesion from the RARβ2-tumor implant was a micro-metastasis in a nude recipient. SCID recipients showed the highest metastatic incidence in the control implants (55%) compared to none in the animals receiving RARβ2-bearing xenografts.

Very few experiments have been conducted to determine the downstream factors regulated by ectopic RARβ2 expression. One such study with F9 teratocarcinoma cells, employing expression microarrays and subtractive hybridization, found differential expression of transcription factors, signalling molecules and metabolic enzymes [18]. Moreover, these investigators found that RARβ2 altered gene expression patterns even in the absence of AT-RA. Toulouse and colleagues transfected RARβ2 into two lung cancer cell lines that lacked endogenous RARβ2 expression [19]. Using the Clontech Atlas human cDNA array, they identified a significant number of genes involved in immune responses. It is likely, however, that unique expression profiles will be found with ectopic RARβ2 expression, depending upon the cell of origin. We used cultured cells, identical to those in the xenograft studies, to conduct expression microarray experiments in order to uncover gene functions or physiologic activities that may prevent metastasis due to overexpression of RARβ2. Due to the hierarchical transcriptional regulation by RARβ in embryogenesis and development, we hypothesized that multiple regulatory pathways, direct or indirect, could be mediated by overexpression of RARβ2 in breast cancer cells.

## Methods

### Cells and xenograft tumors

MDA-MB-435 human breast cancer cells were transduced with either the LXSN-vector or LXSN-RARβ2. Plasmid production, generation of retroviral vectors, and cell culture have been described previously [8,20]. For the microarrays, cells were grown to confluency in single Corning 150 mm cell culture-treated dishes in breast tumor cell media [16]. Two independent culture sets (pairs) were used among the eight arrays. Cells and RNA for validation (below) came from the same lineage (within one passage) as the cells used in the arrays. Tissues were primary, resected tumors from our previous study [8]. Primary xenograft tumors were resected and snap frozen in cryopreservation media in liquid nitrogen. RNA was extracted directly from archived primary tumor tissue. Additional LXSN-vector and LXSN-RARβ2 transductions were performed using the parental MDA-MB-435 cells from a passage near that of the original transduction and cloning. These latter cells were grown under selection (500 μg/mL G-418) on Corning 150 mm cell culture-treated dishes for 10 days when the cells were near confluent, at which time RNA was collected, as below, for qRT-PCR.

### RNA isolation

Total RNA was isolated with Invitrogen's Micro to Midi kit (Carlsbad, CA) according to the manufacturer's protocol for cells or tissue, respectively. The purity and integrity of the RNA was evaluated by measuring the 260/280 nm optical density ratio and either by use of the Agilent Bioanalyzer (Agilent Technologies, Palo Alto, CA) or by denaturing agarose gel as for Northern blots [10].

### RNA labelling, array hybridization, and feature extraction

Five μg of RNA from each transduced cell lineage (LXSN-vector or LXSN-RARβ2) was labelled using the Agilent Fluorescent Linear Amplification kit (Palo Alto, CA). Labelled RNA was further purified using the Qiagen RNeasy Mini kit protocol for liquid samples (Valencia, CA). Four pairs of labelled cRNA were prepared from two independent RNA extractions, hybridized to the Agilent Human 1A Oligo Array, and washed using the Agilent In situ Hybridization Kit Plus. cRNA was labelled with Cy5 and Cy3 for "swapped"-labelling for co-hybridizations on different chips. Hence, every chip had a complimentary chip with a swapped dye configuration, for a total of eight chips. Microarrays were scanned in an Agilent DNA Microarray Scanner and expression data were obtained using the Agilent Feature Extraction software (version 6.1.1), using defaults for all parameters.

### Statistics and analysis

The image file was processed using Agilent's Feature Extraction software. This Feature Extraction program was used to identify pixels corresponding to fluorescent signal (as opposed to background) and to remove pixels with intensities that met the default criteria for outliers. No background subtraction was used nor were any of the available error models used, since the value of these has not been proven and our replicate data were more highly correlated without the use of these features. For each identified area of signal and each of the two dyes, the basic measure of RNA abundance was taken to be the mean intensity over pixels in the identified signal area. The log ratio of the red to green intensities for each signal area (the mean over approximately 60 pixels per oligonucleotide) was used for statistical analyses, with all subsequent analyses done using the R statistical software package [21]. The unit of analysis for the array data was a sample from a single cell line with parallel preparations for all samples and a sample size of eight.

Since the purpose of this study is partially hypothesis formation (as opposed to strict hypothesis testing), we elected *a priori *to examine the 100 most extreme median log ratios (over the eight cell culture arrays) for large differences between the treatments as well as biological patterns that could lead to more explicit hypothesis testing in future experiments using primary xenograft tumors. The median was chosen as the summary measure of expression across the eight arrays (for each oligonucleotide) because the median has been shown to have better performance than mean-based statistics, such as the t-statistic or the SAM statistic, in identifying spiked-in RNAs using small sample sizes [22]. This method of analysis is also similar to that suggested by Breitling et al. [23], with both biological and statistical considerations used to choose the analysis method, except that especially low or high ranks are given less weight in the current method than in the rank product method suggested therein. Before median log ratios were calculated, data from each array were normalized separately using the R function lowess, with a window size of approximately 360 points. The joint significance level (p-value) for finding 100 median log ratios as extreme or more extreme than observed was determined using the permutation test under the null hypothesis of no differential expression in any of the genes between the two groups, as in Pollard and van der Laan, comparing the 100^th ^absolute value order statistic to its permutation reference distribution [24].

Standard binomial probabilities were used to determine the significance of concordance between quantitative real-time PCR (qRT-PCR) results [see below] using RNA from cultures and tissue and the apparent over-representation of Xq28 genes in the top 100 list.

Finally, since an unusually large number of top 100 ratios corresponded to genes on Xq28, we searched the sequence of Xq28 (positions Xq28:146,767,469–154,804,778 from human assembly, hg17, using Known Gene data downloaded from the UCSC Genome Browser [25], for matches to the conserved portions of the RARβ DR5 DNA binding element (βRARE) using the UNIX utility "agrep" [26]. Six βRARE sequences were found within the 89 listed "Known Genes" (exons, introns, plus 1000 upstream and downstream base pairs) on Xq28. In addition, the genomic regions for three other non Xq28 genes of interest (ZADH1, ZNF387, FABP6), including the leading and ending 100 base pairs, were searched for RAREs.

### Validation: quantitative RT-PCR, northern blot and western blot

Total RNA from cells and tissue was treated with 1 U of RNase-free deoxyribonuclease 1 (DNase 1) (Ambion, Austin, TX) per μg of RNA to eliminate genomic DNA contamination. DNase 1 was inactivated with the addition of 2.5 mM EDTA (pH 8.0), followed by 10 minutes at 65°C. cDNA synthesis and PCR analysis were performed using the Invitrogen SuperScript III Platinum Two-Step qRT-PCR Kit with SYBR Green. Quantitative gene expression was detected using the LightCycler (Roche Molecular Biochemicals, Mannheim, Germany). The Invitrogen protocol includes a step using uracil DNA glycosylase and dUTP to prevent amplification of carryover PCR products.

All primers (Supplemental Table 1) were designed with Primer3 [27] using sequences contained in the 60-mer oligonucleotide probes found on the Agilent Human 1A Oligo Array. The arrays contain probes synthesized *in situ *on glass slides by phosphoramidite chemistry. Probes were designed with a 3' bias from collective database sources representing 17,086 genes [28]. Primers were synthesized by Qiagen [aka Operon] (Valencia, CA). qRT-PCR reactions for all samples were carried out in triplicate for each gene. In some cases, additional qRT-PCR was performed with independent RNA samples, as indicated in Tables 1 and 5. Genomic DNA from MDA-MB-435 parental cells was used to generate a standard curve, as this consistently gave values for slope, error and regression coefficient recommended for optimal results by Roche and based on calculations by Pfaffl [29]. All samples were normalized with respect to the constitutively expressed large ribosomal phosphoprotein P0 (RPLP0, aka 36B4) gene. The normalized data were then used to generate a differential expression ratio to compare to the expression microarray data. In cases where we were unable to confirm the expression differential obtained from the microarray analyses, we interrogated Map Viewer [30] or AceView [31] to determine if the 60-mer overlapped mRNA isoforms (e.g., splice variants) known for the gene of interest.

**Figure 1:**
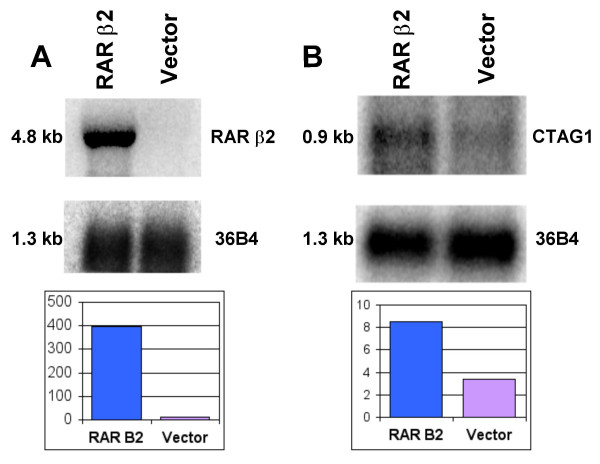
**Expression of RARβ2 and CTAG1 by Northern analysis**. Northern analysis of RARβ2 (**A**) and CTAG1 (**B**) in RARβ2-transduced MDA-MB-435 cells compared to vector-control cells. Using total RNA used in the arrays, Northern blots were probed with ^32^P-dCTP labelled RARβ2 or CTAG1 cDNA. The RARβ2 probe consists of ~1.2 kb KpnI-BamH1 digest fragment of pSG RARβ2 ([75], from Pierre Chambon). The CTAG1 probe was generated from a 463 bp reverse transcriptase PCR product that encompasses the Agilent 60-mer. Blots were sequentially probed with a cDNA for RPLP0 (36B4) as a loading and transfer control. Quantitation of relative transcript levels was normalized to RPLP0 by phosphorimaging using exposure levels within the linear range of detection, avoiding saturation. The RARβ2 mRNA includes elements transcribed from the retroviral vector [8].

Northern blot methods are as described [32]. Northern probe production for SPP1 and CTAG1 is as follows: the 1384 bp SPP1 probe was generated from vector control cells using Qiagen's One step RT-PCR mix (with primers: Fwd CATCACCTGTGCCATACCAG and Rev CCGTGGGAAAACAAATAAGC). The 463 bp CTAG1 probe was generated from both RARβ2 and vector control cells using Qiagen's One step RT-PCR mix (with primers: Fwd CTTCAGGGCTGAATGGATG and Rev AACAAACATGTAAGCCGTCCT). An example of the difference in the level of RARβ2 mRNA in the transduced cells is shown in Figure 1A.

Two independent samples of the RARβ2- and vector-transduced cells were analyzed for SPP1/osteopontin using methods from Tuck et al. [33]. Briefly, 5 × 10^5 ^cells were plated on Corning 100 mm cell culture-treated dishes in breast cancer cell media [32] and incubated for 18 hours. Cells were then grown in 3 ml of serum-free breast cancer cell media for 24 hours. The media was centrifuged briefly to eliminate cellular debris, and then concentrated in an Amicon Ultra-4 (Millipore, Billerica, MA) with a 10 kDa cut off at 1500 rpm for 1.5 hrs, resulting in a final volume of ~60 μL each. The adherent cells were trypsinized and counted using a hemocytometer. Cells were lysed, using a mild lysis buffer (50 mM Tris-HCI pH 8, 300 mM NaCI,10 mM MgCI_2_,1 mM EDTA, 25 mM β-glycerophosphate, 0.5% lgepal-CA 630 [Sigma I3021]) [34] with a protease inhibitor cocktail (Roche, Mannheim, Germany). Protein from ~3 × 10^5 ^cell equivalents of both media and cell lysates were separated, along with recombinant SPP1 (gift from Dr. CM Giachelli), with SDS-PAGE and then blotted to PVDF membrane (BioRad, Hercules, CA). The membrane was probed using a 1:3,500 dilution of a goat anti-SPP1 polyclonal antibody (gift from Dr. CM Giachelli) followed by a horseradish peroxidase-conjugated rabbit anti-goat antibody at 1:20,000 (Pierce, Rockland, IL). The blot was visualized by chemiluminescent detection (Pierce).

## Results & discussion

### Differential expression level changes comparing cells with metastatic potential to RARβ2-overexpressing cells

RARβ2 is overexpressed in MDA-MB-435 cells transduced with a retroviral vector containing the full-length RARβ2 cDNA (Figure 1A). We hypothesized that the antimetastatic functions of RARβ2 were transcriptionally mediated, independent of excess or therapeutic concentrations of ligand, AT-RA, as it has been demonstrated that RARβ2 can modulate gene expression even in the absence of exogenous ligand [18,35].

Of the top 100 candidates (Additional File 2: Supplemental Table 2), which includes rank, fold change (RARβ2/vector), chromosome localization, and URL links to GeneCards), eighty-six comprised unique gene candidates, the remaining being duplicate probes on the arrays. The p-value for finding 100 median ratios this extreme or more extreme was p = 0.004. Fifty-three of the eighty-six unique probes (62%) indicated induced expression in RARβ2-transduced cells.

The relative ratios in expression levels between the RARβ2-transduced cells/vector control cells and the magnitude of the differences were confirmed in fourteen of the top twenty ranked median candidates (70%) by qRT-PCR (Table 1). In addition, CTAG1 expression differences were confirmed by northern blot analysis (Figure 1B). For two of the fourteen confirmed candidates, FABP6 and NNMT, the magnitude detected by qRT-PCR was greater than that detected with the expression microarrays. Overall, there was excellent concordance as to whether the gene candidate was induced or diminished between the array and qRT-PCR results.

Five genes were not confirmed (NC) by qRT-PCR. However, for each of these candidates, we hypothesize that there is potential overlap of an mRNA isoform with the Agilent 60-mer probe, as determined in Map Viewer or AceView for each gene. The remaining single probe, OR52P1, is a pseudogene, which could not be validated.

The top twenty genes obtained from the statistical analysis and subsequent validation provide an interesting window into the antimetastatic cellular functions mediated by RARβ2 in MDA-MB-435 cells. Three candidates, all reduced in RARβ2 cells, are implicated in cell adhesive properties of tumors (LSAMP [rank 1], PCDH11Y [rank 5], and CD164 [rank 8]). CD164 is a sialomucin, and in general, both transmembrane and secreted mucins can regulate growth factor receptor activity. Furthermore, cancer cells may subvert the normal role of the extensively post-translationally modified mucins or sialomucins in order to sense the environment and regulate metastasis [36]. Another category of genes, all with diminished expression in RARβ2 cells, are implicated in nutritional regulation or sensing, and hormone metabolism: SLC38A2 [rank 2], PCSK4 [rank 6], SR-BP1 [rank 11], and FABP6 [rank 12]. Two other genes of note in the top twenty ranked candidates that are elevated in RARβ2 cells are NPDC1 [rank 15] and ZNF387/ST18 [rank 20]. Little has been reported on these genes, but both are likely transcriptional regulatory molecules. NPDC1 has been associated with cessation of proliferation in the nervous system [37], and ZNF387/ST18 is hypothesized to be a tumor suppressor gene in breast cancer [38].

In order to rule out a clonal influence on the antimetasatic phenotype of RARβ2, we also performed qRT-PCR on mixed clones from our original transduction in order to look at a more heterogeneous culture. The pools consisted of equal numbers of cells from five RARβ2 clones and three vector-control clones. RNA extracted from these mixed populations underwent qRT-PCR, using primers from the top seven ranked genes. As shown in Additional File 3: Supplemental Table 3, all seven genes were validated. Additionally, we introduced LXSN-control and LXSN-RARβ2 vectors into MDA-MD-435 cells, and following a ten-day selection in G-418, collected RNA from the newly transduced cell populations. Also shown in Supplemental Table 3, six of the seven top genes were validated.

### Genes in the cancer/testis antigen family localized to Xq28 are elevated in RARβ2 overexpressing cells

Eleven of the top 100 (87 unique) genes reside on the X chromosome, including CTAG1 [rank 3] (Supplemental Table 2 & Table 2). Of these, eight map to Xq28, the most telomeric cytogenetic long-arm band (Table 2). Given that there are seventy-five Xq28 genes on the array, p = 2 × 10^-8 ^for finding eight Xq28 genes among the most differentially expressed 86 unique genes, using an *a priori *null hypothesis of no preference for Xq28 genes. Five of the eight probes or genes belong to the large cancer/testis (C/T) antigen gene family, which include CTAG1, CTAG2, LOC389903, MAGEA2, and TRAG3. The array experiments suggested that the C/T genes are induced either directly or indirectly by the presence of RARβ2 compared to the empty vector cells. We originally evaluated and validated CTAG1 by Northern blot analysis (Figure 1B) and secondarily by qRT-PCR, both of which showed an approximately two-fold or greater expression in RARβ2-transduced cells/vector control cells. The mRNAs for two C/T genes, LOC389903 and TRAG3, share significant sequence overlap within their respective 60-mer probes, which may account for our inability to confirm expression rations for these genes by qRT-PCR. Similarly, the MAGEA2 60-mer probe differs by 1 or 2 nucleotides compared to MAGEA3, MAGEA6, or MAGEA12.

C/T genes consist of over forty families, and their expression may not be limited to germ line cells as a small subset are expressed in somatic cells, particularly the pancreas [39,40]. As discussed by Scanlon et al., many of these proteins are expressed in breast cancers [40]. Recently, Montel et al. performed expression profiling using two well-characterized clones derived from MDA-MB-435 cells, one that metastasized in xenograft experiments, and the other that failed to metastasize. They found that a number of C/T genes (including CTAG1, CTAG2, a colon cancer antigen 16, and MAGEA1) were elevated in the clone that did not metastasize [41]. Theoretically, an adaptive immune response may be responsible for biologic effect; however, members of the C/T family may have as yet unknown functions, independent of tumor antigenicity. For example, MAGE-11 was recently shown to be a co-activator of the androgen receptor in androgen dependent tissue. This latter discovery was conducted with yeast two-hybrid interactions and functional analysis [42]. Cronwright and colleagues found that the C/T genes are expressed in mesenchymal stem cells but are down-regulated following differentiation [43]. This latter observation supports the interesting hypothesis that cancer cells are stem cell derivatives.

### Additional genes on Xq28

Three additional genes on Xq28 with apparent up-regulation by RARβ2 were confirmed by qRT-PCR (Table 2). ARHGAP4 is a GTPase-activating protein that may modulate stress fiber organization and function [44]. RPL10 is a 60S ribosomal protein that may possess other functions, including the ability to bind cYES kinase and affect SH3-mediated signal transduction cascades, resulting in tumor suppressor functionality [45]. Finally, SSR4, signal sequence receptor delta 4, was also elevated in RARβ2 cells. SSR4 encodes a protein subunit for a complex involved in protein secretion [OMIM:300090].

### Is Xq28 enriched with beta retinoic acid response elements?

Six potential RAR binding elements were found during the search of the genomic coding sequences, including 1000 base pairs upstream and downstream from the start and stop codons, corresponding to known genes on Xq28. In this analysis, we simply searched for previously reported RARβ response elements [46], since this has the potential to provide additional information regarding regulatory pathways. Additional File 4: Supplemental Table 4 lists the sequences found and their locations. In comparison, no known RAREs were found in the regions of ZADH1, ZNF387, nor FABP6, none of which are Xq28 genes, though this does not rule out the possibility that novel RAREs or more distant RAREs contribute to the expression of these genes. These are intriguing results, given the large number of Xq28 genes with differential expression in this experiment. More research is needed to determine the significance of these results, both statistically (after determining the appropriate control for comparison of the number of elements found) and biologically (by testing of authentic RARβ2 binding within the larger context of the sites). We note that the genes in Supplemental Table 4 were all on the array but were not in top 100 ranked genes (Supplemental Table 2). One caveat to consider is that even if the putative RAREs are nearest to the noted genes, the RARE sites could act as distant transcriptional regulatory elements, as is the case for the HOX gene cluster, where RARE activation may occur at distances from 6 to more than 20 kilo bases [47]. In addition, RAREs may be found in 3' regions of genes [48]. If future expression profiling experiments using tumors should reveal more genes at Xq28, we could test as a control the possibility of retroviral integration at this site by fluorescence in situ hybridization and molecular mapping. Despite the preliminary nature of these results, they provide a basis for further research by us and others as well as demonstrate a potentially simple and powerful auxiliary analysis to array experiments that makes use of the growing amount of knowledge of consensus sequences.

### Candidate genes involved in immune response and interferon signalling

As introduced above with the C/T genes on Xq28, a high proportion of the most extreme 100 median-log genes are associated with immune function. Table 3 lists additional immune function candidates. Of the nine genes in Table 3, five are known to be regulated through interferon or are involved in interferon signalling: IFI27 [rank 81], IFIT1 [rank 47], IFITM1 [rank 28], RIG-I [rank 79], and USP18 [rank 35].

Two genes, IFIT1 and IFITM1, are elevated in RARβ2 cells compared to control cells. IFIT1, which is induced by interferon alpha in response to viral infection, contains ten tetratricopeptide repeats, and these repeats are hypothesized to confer stability to the protein [49]. IFITM1 is induced by both alpha and gamma interferons. Based on the protein sequence, IFITM1 is likely an integral membrane protein, and exogenous expression in COS cells confers an antiproliferative, non-migrating phenotype [50]. A recent study of chronic myelocytic leukaemia patients determined that expression of IFITM1 in neoplastic cells predicted improved survival [51].

Two other interferon regulated genes are diminished in RARβ2 cells compared to control cells, IFI27 and USP18/UPB43. In one study, ~50% of human breast cancers expressed elevated IFI27 RNA, which was inversely correlated with estrogen receptor status [52]. Moreover, retinoids have been shown to suppress IFI27's expression [53]. Interferon signalling pathways are multi-nodal and involve molecules such as AKT, PCK, various STATs and NFκB [54]. The results of our expression microarray studies suggest that exogenous RARβ2 may modulate interferon signalling in MDA-MB-435 cells, leading to enhanced immune surveillance 'in the host.' Expression profiles indicated that RIG-I/DDX58 [rank 79] was elevated in RARβ2-transduced cells. This observation was confirmed with qRT-PCR for both the cell cultures (Table 3) and tumor tissue (Table 5). RIG-I is a retinoic acid inducible gene that encodes an RNA helicase/adaptor protein that is critical in sensing virus and propagating interferon gamma innate responses [55].

### Candidate genes involved in transcription regulation

RARs are requisite transcription factors for a vast array of biological functions, such as embryogenesis, development, homeostasis, vision, and the immune system [56]. There are likely thousands of genes regulated directly through RAR transcriptional activation through canonical binding elements in gene promoters. One of the more well-characterized systems in development is the interplay between RARs and HOX gene expression [57]. Table 4 lists candidate genes that may have a transcriptional function in either permitting or abrogating metastasis. HOXB7, which has higher expression in the control cells, may have a role in neoplasia, as it was shown to block differentiation of myelocytic cells [58]. ZNF387/ST18 (see also Table 1) likely exhibits tumor suppressor function of as yet unknown mechanism.

The finding of a potential down-regulation of the cJUN mRNA is very intriguing, as RARs and members of the AP1 family such as JUN have major antagonistic interactions and both gene families are master regulators of many physiologic functions. JUN and AP1-associated family members are key transcription factors that block the antiproliferative functions of p53, p21, and p16 [59]. The mechanisms of cross-repression by RARs [60], and particularly by RARβ2 [61], which inhibit JUN and AP1 activity, include sequestering of co-activators and blockage of signalling pathways that recruit transcriptional activators such as CBP [62]. We demonstrated metastasis suppression in the absence of pharmacologic ligand, AT-RA [8]. Moreover, it has been independently demonstrated that RARβ can abrogate AP1 activity and inhibit anchorage independent colony formation of RARβ-stably transfected MDA-MB-231 breast cancer cells [63]. Two other gene candidates apparently regulated by the transcription factor NFκB, SECTM1 [Table 1, Rank 17] and SLC20A1 [Rank 29], are also elevated in RARβ2 cells, and NFκB signalling is likely an important pathway mediating metastasis suppressor functions, including RIG-I [7,64].

### A cautionary note concerning unexpected findings in gene array validation

**Figure 2:**
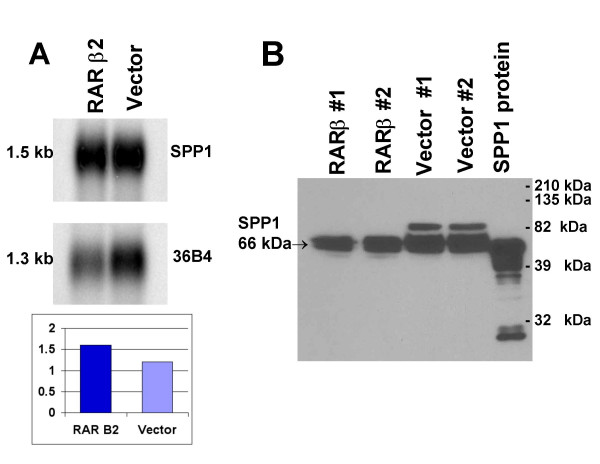
**SPP1/Osteopontin expression and a validation dilemma**. SPP1 expression comparison of RNA and protein. A. Northern blot analysis. Eight μg of whole cell total RNA extracts were probed with a 1384 bp fragment of SPP1 (see methods) or 800 bp, *Pst1 *fragment of RPLP0 (36B4). Phosphorimaging was used for detection and quantitation. B. Western immunoblot analysis. Two independent preparations of each clonal cell line were used. Three mL of serum free-media from 3 × 10^5 ^cell equivalents was immunoblotted, using a goat anti-SPP1/osteopontin polyclonal antibody (antibody and recombinant protein were a gift from Dr. CM Giachelli) and a secondary rabbit anti-goat antibody (Pierce).

Ten of the top 100 gene candidates were SPP1 (osteopontin), and there were ten independent SPP1 probes of the same sequence on the Agilent arrays (Supplemental Table 2). The statistical results indicated that SPP1/osteopontin was elevated (~1.4 fold) in RARβ2-transduced cells. We carefully investigated this unexpected finding, as SPP1/osteopontin may be a key player in promoting metastasis [65] or breast cancer progression [66]. Moreover, SPP1/osteopontin may be a critical protein component in breast cancer's ability to metastasize to bone [67]. Northern blot analysis also suggested that SPP1/osteopontin was marginally elevated at the steady state level in RARβ2 cells (Figure 2A). qRT-PCR findings also showed an increase in the level of SPP1/osteopontin message comparing RARβ2- to vector control-cells. Finally, we analyzed the media of the vector control and RARβ2 cells by western blot for possible differences in protein levels. Interestingly, we detected both slightly higher protein levels plus a higher molecular weight form of SPP1/osteopontin protein in the vector control cells. There was no evidence for SPP1 protein in the western blot of cellular lysates, only from the concentrated media. (Figure 2B). SPP1/osteopontin can be post-translationally modified via a vast variety of moieties, including N- or O-linked glycosylation, phosphorylation, cross-linking with other proteins, deamidation, oxidation and carbamylation. Recently mass spectrometry experiments demonstrated that SPP1 has over thirty potential phosphorylation residues [68,69]. Whatever the sum of post-translational changes, the modified protein is likely a better substrate for possible interactions with fibronectin that may confer enhanced cellular migration.

### Comparison of candidates derived from cell culture with primary xenograft tumors

We evaluated expression of selected genes of interest in two pairs of xenograft tumors from nude mice. We chose the gene candidates using the following criteria: 1) they had been validated by qRT-PCR in the cell cultures; and 2) they sampled the full rank range (Table 5). One candidate, CA14 [carbonic anhydrase 14, rank 86], with evidence for RARβ2-mediated diminished expression, is elevated in a variety of cancers, particularly hypoxic tumors [70]. Another candidate, NDRG1 [N-Myc down-stream regulated gene 1, rank 42], is regulated by the PTEN protein and may control metastasis in colon, prostate, and breast cancers [71,72]. Although the precise function(s) for NDRG1 has not been elucidated, Kim et al. demonstrated p53-dependent microtubule check point function for the protein in breast cancer cells [73]. Stein et al. found that NDRG1 was transcriptionally regulated by p53 and is necessary, but not sufficient, for p53 modulated apoptosis [74]. Thirteen of fifteen selected genes are consistent between cell cultures and the randomly selected tumors (p = 0.007). Thus our hypothesis testing, using cell lines from the xenograft studies, suggests that RARβ2-manifest anti-metastasis functions preexist in the transduced cells.

## Conclusion

**Figure 3:**
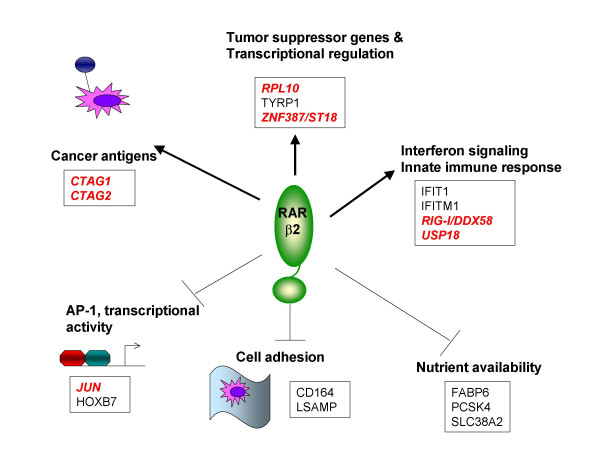
**Model of antimetastatic functions modulated by RARβ2**. Expression of RARβ2 in metastatic breast cancer cells confers multiple antimetastatic properties, including induction of cancer antigens, tumor suppressors, and genes involved in interferon signalling. Other gene activities are suppressed through RARβ2 action: AP1, cell adhesion, and nutrient processes. All gene activities in this diagram have been confirmed by cell culture qRT-PCR. Gene names in italics (red), have also been confirmed using randomly selected xenograft primary tumors, comparing two pairs of vector control- and RARβ2-resected tumors.

Our expression profiling experiments revealed both potential activated and repressed cellular activities in response to overexpression of RARβ2 (Figure 3). RARβ2 induces expression of several members of the C/T antigen family found on Xq28 as well as interferon signalling genes (Tables 2 and 3), suggesting that therapeutic modulation of RARβ2 in tumor cells may enhance endogenous immunity or potentially be additive to therapeutic immunomodulation. Our profiles indicate that RARβ2 induces a number of tumor suppressor functions (NDRG1, RPL10, and ST18) and known metastasis suppressors (NDRG1 and TYRP1). We find that the expression of a number of genes involved in cell adhesion (LSAMP, PCDH11Y, and CD64); nutrient availability (FABP6, SLC38A2, PCSK4, SRPB1); and transcription/AP1 activity (HOXB7 and JUN) are repressed in RARβ2 cells. Our findings suggest new arenas of complimentary or synergistic pathways in the regulation of metastasis.

## Competing interests

The author(s) declare that they have no competing interests.

## Authors' contributions

KS, BW, and ME designed the project. BW performed the microarray and validation experiments. ME performed statistical analysis and consensus sequence matching. MES performed the genome annotation for response elements on Xq28. MLD reviewed and contributed to the discussion of the immune function data. All authors contributed to the writing of the manuscript and have read and approved its revised and final drafts.

## Pre-publication history

The pre-publication history for this paper can be accessed here:



## Supplementary Material

Additional File 2 Supplemental Table 2 – Curated top 100 median log ratios Click here for file

Additional File 3 Supplemental Table 3 – Comparison of expression of pooled clones, individual clones, and additional short term transduction Click here for file

Additional File 4 Supplemental Table 4 – Binding elements detected on chromosome Xq28 Click here for file

Additional File 1 Supplemental Table 1 – Oligonucleotide sequences of primers for qRT-PCR Click here for file

## Figures and Tables

**Table 1:** Top twenty ranked genes

**Gene Symbol (Probe)**	**Name**	**†Fold change (qRT-PCR)**	**Rank**	**Function/Other information**
LSAMP	Limbic system associated membrane protein	-3.2 (-3.1)	1	Immunoglobulin superfamily member; cell adhesion
SLC38A2	Solute carrier family 38	-2.2 (-2.1)	2	Amino acid transporter A2, regulated by growth factors & hormones
CTAG1	Cancer/testis antigen 1	2.2 *(1.6, 1.5)	3	(see also Table 2)
MGC2780	Hypothetical protein	-2.2 (-5.5)	4	Unknown
§PCDH11Y	Proto-cadherin on the Y	-2.1 (-1.9)	5	Cell adhesion X chromosome homologue
PCSK4	Convertase; subtilisin/kexin 4	-2.0 (-1.6)	6	Cleave pro-hormones and pro-growth factors
ZADH1	Zinc-binding alcohol dehydrogenase domain containing protein	1.9 *(1.3, 1.2)	7	Alcohol dehydrogenase; possible retinoid metabolism
CD164	Cluster designation protein 164	-1.9 (-1.5)	8	Sialomucin, AKA, MGC-24
SFTPA2	Surfactant pulmonary associated protein	1.8 NC	9	Calcium dependent signalling innate immune response
FLJ23577	Hypothetical Kruppel-like protein	-1.8 NC	10	unknown
SR-BP1, OPRS1	Sterol binding protein Opoid receptor sigma	-1.7 NC	11	Sterol isomerase, ergosterol biosynthesis
FABP6	Fatty acid binding protein 6	-1.7 (-6.6)	12	Bile acid binding;
OR52P1	Olfactory receptor 52 pseudogene	1.7 NC	13	Pseudogene
CSTA	Cystatin A	-1.7 (-2.3)	14	Cysteine proteinase inhibitor
NPDC1	Neural differentiation & control protein	1.7 *(1.5, 1.3)	15	Inhibits proliferation
NXT2, P15-2	Nuclear transport factor 2-like export factor	1.7 NC	16	Exports RNA from nucleus
SECTM1	Secreted and transmembrane protein 1	-1.6 (-2.8)	17	Signal transduction activity with the NF-κB cascade
HKE2	HLA class II region KE2 gene	1.6 NC	18	Protein folding
NNMT	Nicotinamide N-methyltransferase	-1.6 (-4.6)	19	Methylates pyridines; biotransformation of drugs; enhanced in cancer cells
ZNF387, ST18	Zinc finger protein 387; breast cancer tumor suppressor	1.6 (2.7)	20	Transcription (see also Table 4)

† "Fold change," top number is the relative expression of RARβ2 transduced cells/empty vector control cells from Agilent arrays. The number in parentheses is the relative expression of the same cells from qRT-PCR analysis, normalized by respective qRT-PCR RPLP0 expression.

NC = not confirmed. In all cases, these genes have known isoform(s) [with exception OR52P1, which is a pseudogene] that overlap(s) the 60-mer probes on the arrays, which could account for the discrepancy between array finding and qRT-PCR.

* two qRT-PCRs (each in triplicate) of independent RNA samples

§shares near identical homology to homologue on X chromosome

**Table 2:** Gene cluster on Xq28

**Gene Symbol (Probe)**	**Name**	**†Fold change (qRT-PCR)**	**Rank**	**Function/Other information**
ARHGAP4	Rho-GAP GTPase	1.4 (2.2)	44	Possible inhibitory role on stress fiber organization and microtubules
§CTAG1	Cancer/testis antigen 1	2.2 (1.6)	3	
§CTAG2	Cancer/testis antigen 2	1.3 (1.6)	93	
§ *LOC389903	Cancer antigen	1.3 (NC)	75	
§ **MAGEA2	Cancer antigen	1.3 (NC)	73	
RPL10	Ribosomal protein	1.6 (1.4)	22	Tumor suppressor
SSR4	Signal sequence receptor/translocon associated protein delta	1.4 (1.2)	52	Type I receptor in endoplasmic reticulum/intracellular protein transport
§ *TRAG3	Tumor antigen	1.3 (NC)	77	

† "Fold change," top number is the relative expression of RARβ2 transduced cells/empty vector control cells from Agilent arrays. The number in parentheses is the relative expression of the same cells from qRT-PCR analysis, normalized by respective qRT-PCR RPLP0 expression.

NC = differential expression was not confirmed by qRT-PCR

§ Genes in the cancer/testis antigen superfamily

* Shares significant overlapping sequence within their respective 60 mer probes, differing by 2–3 nucleotides

** MAGEA2 60 mer probe shares significant sequence identity with MAGEA-3, -6, and -12 (differs by 1–2 nucleotides)

**Table 3:** Immune response and interferon signalling

**Gene Symbol (Probe)**	**Function/family**	**†Fold change (qRT-PCR)**	**Rank**	**Function/Other information**
APP	Amyloid precursor protein	1.6 (ND)	21	Proinflammatory
HLA-DRB1	Histocompatability complex, class II, DR beta 1	-1.3 (ND)	89	Signal transduction; antigen processing
IFI27	Interferon (IFN)-alpha inducible Protein 27	-1.3 (-1.9)	81	Integral membrane protein; unknown function
IFIT1	IFN-induced protein with tetratricopeptide repeats	1.4 (1.3)	47	Transmembrane protein; Interferon, retinoic acid inducible
IFITM1	IFN-induced transmembrane protein 1	1.5 (2.9)	28	Growth inhibition
PTMA/TMSA	Prothymosin A	-1.4 (ND)	53	Innate immunity; nuclear; associated with proliferation
RIG-I/DDX58	Retinoic acid inducible gene I/DEAD box 58	1.3 (1.7)	79	IFN signalling; RNA helicase
TRA1/GP96	Tumor rejection antigen 1	1.3 (NC)	98	Glycoprotein; stress response
USP18/UBP 43	Ubiquitin specific protease 18	-1.5 (-1.6)	35	Protease/ubiquitinase regulated by IFN

† "Fold change," top number is the relative expression of RARβ2 transduced cells/empty vector control cells from Agilent arrays. The number in parentheses is the relative expression of the same cells from qRT-PCR analysis, normalized by respective qRT-PCR RPLP0 expression.

ND = not done

NC = differential expression was not confirmed by qRT-PCR, and this gene has mRNA isoform that overlaps the 60-mer probe

**Table 4:** Transcriptional regulation: global and targeted

**Gene Symbol (Probe)**	**Name**	**†Fold change (qRT-PCR)**	**Rank**	**Function/Other information**
ANC 2H01/ZNF639/ZASC1	Zinc finger protein 639	1.3 (ND)	76	Kruppel domain
CITED4	CPB/p300-binding protein & interacting 4	1.3 (ND)	74	Transcriptional coactivator
FJL23577	Hypothetical, Kruppel-like protein	-1.8 (NC)	10	Unknown
HIPK2	Homeodomain-p53-interacting protein	-1.4 (ND)	51	Homeodomain interacting protein kinase 2
HOXB7	Homeobox gene B7 (ANTP family)	-1.4 (-1.2)	38	Inhibits differentiation
*JUN	cJUN	-1.3 (-1.5)	90	AP1 complex
KIAA115	SIT4 phosphatase-associated protein-like	-1.3 (ND)	82	Cyclin/G1 transcription
MGC15737/TCEAL3	Transcription elongation factor A-like 3	1.3 (ND)	100	Transcriptional elongation
ZNF387, ST18	Zinc finger protein 387; breast cancer suppressor of tumorigenicity	1.6 (2.7)	20	Transcription/Tumor suppressor (see also Table 2)

†"Fold change," top number is the relative expression of RARβ2 transduced cells/empty vector control cells from Agilent arrays. The number in parentheses is the relative expression of the same cells from qRT-PCR analysis, normalized by respective qRT-PCR RPLP0 expression

ND = not done

NC = not confirmed, has at least two isoforms that overlap the 60-mer probe

* "cross-talks" with RARs

**Table 5:** Comparison of expression: xenograft tumors and cultured cells

**Gene symbol (Rank)**	**Array fold change**	**Cell fold change**	**†Tissue fold change**
ARGAP4 (44)	1.4	2.2	1.1
CA14 (86)	-1.3	-1.9	-1.2
CTAG1 (3)	2.2	1.6	2.2
CTAG2 (93)	1.3	1.6	1.2
IFI27 (81)	-1.3	-1.9	-1.4
‡IFIT1 (47)	1.4	1.3	-1.2
‡IFITM1 (28)	1.5	2.9	-1.5
JUN (90)	-l.3	-1.5	-1.2
NDRG1 (42)	1.4	4.5	2.9
RPL10 (22)	1.6	1.4	1.2
RIG-I (79)	1.3	1.7	1.1
RNF28 (37)	-1.5	*-2.2/-1.7	-1.4
SSR4 (52)	1.4	1.2	1.6
ST18 (20)	1.6	2.7	1.8
USP18 (35)	-1.5	-1.6	-1.3

†Fold change for tissue was determined by dividing the normalized (to RPLP0) qRT-PCR value of two tumors that contained exogenous RARβ2 by the normalized value of two tumors that contained the empty vector.

‡Map Viewer or AceView indicate that there are overlapping mRNA isoforms with the 60 mer probe.

*two qRT-PCRs (each in triplicate) of independent RNA samples
