# Supplementary material for: Antimetastatic gene expression profiles mediated by retinoic acid receptor beta 2 in MDA-MB-435 breast cancer cells
Source: BMC Cancer. 2005 Oct 28;5:140. doi: 10.1186/1471-2407-5-140 (PMC1283145; doi:10.1186/1471-2407-5-140)
Supplement: Additional File 3 — Supplemental Table 3 – Comparison of expression of pooled clones, individual clones, and additional short term transduction [file 1471-2407-5-140-S3.pdf]

Supplemental Table 3: Comparison of expression of pooled clones, individual clones, and additional short-term transductions

Gene symbol (Rank)	Array fold change	†qRT-PCR of clones	†qRT-PCR of pooled clones	†*qRT-PCR of primary transduced cells
LSAMP (1)	-3.2	-3.1	-2.1	-1.3
SLC38A2 (2)	-2.2	-2.1	-1.3	-1.3
CTAG1 (3)	2.2	1.6	1.2	2.1
MGC2780 (4)	-2.2	-5.5	-1.8	1.7
PCKH11Y (5)	-2.1	-1.9	-8.4	-1.3
PCSK4 (6)	-2.0	-1.6	-1.4	-1.2
ZADH1 (7)	1.9	1.3	1.3	1.2

† Fold changes were determined by dividing the normalized (to RPLP0) qRT-PCR value of pooled clones containing either RARβ2 by the normalized value of pooled clones that contained the empty vector.

* A new transduction of parental MDA-MB-435 cells (6.0 x 10^5) was performed with LXSN-vector or the LSXN-RARβ2. After a ten-day selection, the population was analyzed for gene expression. We note that the one gene that did not show concordance with the cell clones or the pooled clones was MGC2780. This Agilent probe [GenBank:NM_025266] was recently removed from the NCBI database.
