# Supplementary material for: Antimetastatic gene expression profiles mediated by retinoic acid receptor beta 2 in MDA-MB-435 breast cancer cells
Source: BMC Cancer. 2005 Oct 28;5:140. doi: 10.1186/1471-2407-5-140 (PMC1283145; doi:10.1186/1471-2407-5-140)
Supplement: Additional File 4 — Supplemental Table 4 – Binding elements detected on chromosome Xq28 [file 1471-2407-5-140-S4.pdf]

Supplemental Table 4: Binding elements detected on chromosome Xq28

Sequence	Gene	Location	Genomic Architecture
GGGTCA(AGTGG)AGTTCA<br>GGTTCA(AGATCA)AGTTCA<br>GGTTCA(AGATCA)AGTTCA<br>GGTTCA(TATTTT)AGTTCA	**FMR2**<br>Fragile X mental retardation 2	Between exons 3 & 4	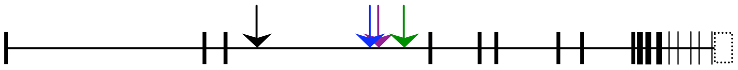<br>147,287,685 - 147,787,753
http://www.ncbi.nlm.nih.gov/entrez/query.fcgi?db=gene&cmd=retrieve&dopt=default&list_uids=2334			
AGTTCA(GGAAA)AGTTCA	**CXorf6**<br>Chromosome X open reading frame 6	Between exons 4 & 5	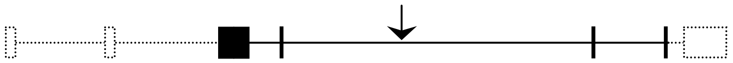<br>149,284,288 - 149,353,007
http://www.ncbi.nlm.nih.gov/entrez/query.fcgi?db=gene&cmd=Retrieve&dopt=Graphics&list_uids=10046			
AGTTCA(CAGAC)AGTTCA	**GAB3**<br>GRB2-associated binding protein 3	Between exons 8 & 9	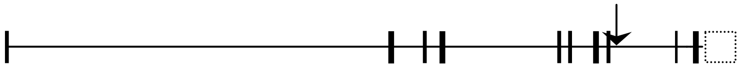<br>153,543,036 - 153,467,235
http://www.ncbi.nlm.nih.gov/entrez/query.fcgi?db=gene&cmd=Retrieve&dopt=Graphics&list_uids=139716			

Arrows indicate approximate location of binding elements. Dotted lines indicate untranslated regions. Nine-digit numbers specify chromosomal locations (start-end) of the genes.

URL to Entrez Gene link provided.
