# Supplementary material for: Antimetastatic gene expression profiles mediated by retinoic acid receptor beta 2 in MDA-MB-435 breast cancer cells
Source: BMC Cancer. 2005 Oct 28;5:140. doi: 10.1186/1471-2407-5-140 (PMC1283145; doi:10.1186/1471-2407-5-140)
Supplement: Additional File 1 — Supplemental Table 1 – Oligonucleotide sequences of primers for qRT-PCR [file 1471-2407-5-140-S1.pdf]

Supplemental Table 1: Oligonucleotide sequences of primers for qRT-PCR

Rank	Gene	Primers	Product Length	Tm
1	LSAMP	Fwd GAGAGAGAGAGAGAGAGAGAGATGG<br>Rev AAAGTTGTGAAATAAACGGTCTCC	50	59-60
2	SLC38A2	Fwd TCTCGGGTTAAGGGAAGTGA<br>Rev CATTCTCTGGAATGGAATAAAATTG	45	60
3	CTAG1	Fwd TTGTCGCTGGAGGAGGAC<br>Rev TTTTCTACAGAAACAAACATGTAAGC	45	58-60
4	MGC2780	Fwd GAGAGAGAGATAGAATTCCAGCAG<br>Rev CCATTCGTGGAAATGTGTACTT	50	58-59
5	PCDH11Y	Fwd TTATGACCATTAGAAACTATTTTGA<br>Rev AATTGAGCCAGTTGGAAAC	46	54-55
6	PCSK4	Fwd GAAGTCTCCTCTGCATTTTGG<br>Rev CACTCCCGCCCAAACC	36	59-61
7	ZADH1	Fwd ATGGGAGCTGCATTCCAGT<br>Rev CTGCTTTCCAATGTTACCTCCT	51	60-61
8	CD164	Fwd GAGTGCTGTAGGATTAATTCGAAAAT<br>Rev GGGAGGAATGGAATTCTGC	47	59-60
9	SFTPA2	Fwd AAGCCACACTCCACGACTTC<br>Rev CCCTTGTCTGCAGGATTTG	45	59-60
10	FLJ23577	Fwd TGGCTGTTGGAACTCATGTC<br>Rev TTGGAATGGAAGCGTTGACT	48	60-61
11	OPRS1	Fwd TTGAAGTCAGCGTCTTCCATT<br>Rev CAAAGGAAGAATGGGGGTCT	47	60
12	FABP6	Fwd CAAGGAAAGCAACATACAGACAA<br>Rev ACAGTGGCCTTGAACGTCTT	51	59-60
13	OR52P1	Fwd ACGTTCCAGTCCATATTCACATT<br>Rev GGTGGCAAAAGCAGATAAACA	55	60
14	CSTA	Fwd TGAGACTTACGGAAAATTGGAA<br>Rev CAACAACTTGAGTTTTATACTGCACA	50	59- 60
15	NPDC1	Fwd GCATGGAGAACCGTAGAGC<br>Rev TGGCTTTTGTCTCTAGAATTACCC	55	58-60
16	NXT2	Fwd TGCTCAGTCCACTCCCAAC<br>Rev TCACTTGCAATCTTCCACACA	45	60
17	SECTM1	Fwd CCTCCTAGAACCCCAGATGAA<br>Rev GGCTCCCGCTCTGAGG	46	60-61

18	HKE2	Fwd GGGAAGAGGCTGGACTATATCA<br>Rev GCTGGGATTCGTATCGCTTA	52	60
19	NNMT	Fwd TGTGAAAGAGGCTGGCTACARev<br>TTTGCGAGATCACCTCAAAC	50	59- 60
20	ST18	Fwd TTCCACTCCCATGAAACTTTG<br>Rev GCCCCACTTAATGCATAACAA	46	60
22	RPL10	Fwd GGCCAAGTTCAAGTTTCCTG<br>Rev ACTTCTTTGAGATGTGGATCTTCTG	50	60
28	IFITM1	Fwd CAGCAGTTTATACCCACACACC<br>Rev GCACGTGCACTTTATTGAATG	55	59
35	USP18	Fwd TCATTTTCCATTTCCGTTCC<br>Rev GCAAAATCTCTTAGAAGACTCCGTA	51	60
37	RNF28	Fwd ACGATGTGCTGTATTTCAGTGTCT<br>Rev TCAGTTACCACCCCGTATGTC	51	60
38	HOXB7	Fwd ACATGAGAAAGGGAGACGAAGA<br>Rev CCTGATTCAGTTCCCAGAGC	50	60
39	MRPL52	Fwd GAGGCTTGGCTTAGTACTCTCATC<br>Rev TTACCTGAACTGGAATGGAACC	49	60
40	SPP1	Fwd CCACAGCCATGAATTTCACA<br>Rev TTGGGGTCTACAACCAGCAT	51	60-61
42	NDRG1	Fwd GCCAGGTTCCTGTACTACTGC<br>Rev ATCCACGGTGAGCCAAAAT	52	59-60
44	ARHGAP4	Fwd TCTGCAGCTTTGTGGAAGG<br>Rev CCACCCAGCAGCCACT	41	60
47	IFIT1	Fwd CTTTGAGAACTCTGTGAGACAAGG<br>Rev GGCTGATATCTGGGTGCCTA	48	60
48	CYP4Z1	Fwd ATCAAAACTCCACTCAGTATCTGC<br>Rev GCAGATATTTGCAGAGATAAAAGTAA	51	58-59
49	PABPC3	Fwd CTTCTTTATATGCTCGAGTCTCC<br>Rev TCATCAACCTTAGAACGGAGTG	50	57-59
52	SSR4	Fwd CTCAGGAAGGCTCAGAGGAA<br>Rev TAAACAGAGGCGGGATGATG	55	60
55	TYRP1	Fwd AAACTTTGGAGAGGGAAAATCT<br>Rev CACAGGCAATATCCATTGTTG	54	57-59
65	TPD52L1	Fwd GCATGTCCTTGAAGGCTGA<br>Rev CTGCTGAACTGAAAGGGACTG	45	60
66	RPL28	Fwd GCAATTCCTTCCGCTACAAC<br>Rev CCACAGTCTTGCGGTGAAT	45	60

72	CD19	Fwd GCCAGCCTGGACCCAAT<br>Rev TAAGAGTCTGCATCTTCCTCATGATT	40	62
73	MAGEA2	Fwd GCAGTCAGCATTCTTAGCAGTG<br>Rev TCAAAGTCATCCAACAGAACAGA	49	60
75	LOC389903	Fwd AACCCCAGATAAATAGCCAACA<br>Rev CTCTTTGGGGTGTAAAATGTACTCT	52	59-60
77	TRAG3	Fwd AACCCCAGATAAATAGCCAACA<br>Rev CCCTTTGGGGTGTAAAACG	54	60
81	IFI27	Fwd CCAGCGAGGAGCCAACTAT<br>Rev ATTTGGTATATTTCACCCCAGGT	51	60
90	JUN	Fwd AACTCATGCTAACGCAGCAG<br>Rev CCGACGGTCTCTCTTCAAAA	49	59-60
93	CTAG2	Fwd GGTGTCGCCTTTAATGTGATG<br>Rev GCTAAATGTGAGGGGCAGAG	43	60
95	NANS	Fwd TAGTGGGCAAGAAGGTCCTG<br>Rev TGATGGTGTCATCCTCTTCAAC	48	60
96	SDF2L1	Fwd TTGGCAGAGACTTTGGGTTT<br>Rev AGGCACTTGAGGACCCCTA	40	60
98	TRA1	Fwd CCTTCTCCCCTGCACTGTAA<br>Rev TGTGACCCATAATCCCACATT	41	60
99	retSDR4	Fwd TCACAAAGACTATAGGCAATAATGGA<br>Rev TTGTCATCTTGTGGGACTGG	48	60
NA	RPLP0	Fwd CTGTTGCATCAGTACCCCATT<br>Rev TCGTTTGTACCCGTTGATGA	44	60
